# The Improved Method for Indoor 3D Pedestrian Positioning Based on Dual Foot-Mounted IMU System

**DOI:** 10.3390/mi14122192

**Published:** 2023-11-30

**Authors:** Haonan Jia, Baoguo Yu, Hongsheng Li, Shuguo Pan, Jun Li, Xinjian Wang, Lu Huang

**Affiliations:** 1 School of Instrument Science and Engineering, Southeast University, Nanjing 210096, China; 230219409@seu.edu.cn (H.J.);; 2 State Key Laboratory of Satellite Navigation System and Equipment Technology, The 54th Research Institute of China Electronics Technology Group Corporation, Shijiazhuang 050081, Chinahlcetc54@163.com (L.H.)

**Keywords:** pedestrian navigation, dual-foot, inequality constraint, Kalman filter, Inertial Measurement Unit (IMU)

## Abstract

Micro-Electro-Mechanical System (MEMS) inertial sensors, characterized by their small size, low cost, and low power consumption, are commonly used in foot-mounted wearable pedestrian autonomous positioning systems. However, they also have drawbacks such as heading drift and poor repeatability. To address these issues, this paper proposes an improved pedestrian autonomous 3D positioning algorithm based on dual-foot motion characteristic constraints. Two sets of small-sized Inertial Measurement Units (IMU) are worn on the left and right feet of pedestrians to form an autonomous positioning system, each integrated with low-cost, low-power micro-inertial sensor chips. On the one hand, an improved adaptive zero-velocity detection algorithm is employed to enhance discrimination accuracy under different step-speed conditions. On the other hand, considering the dual-foot gait characteristics and the height difference feature during stair ascent and descent, horizontal position update algorithms based on dual-foot motion trajectory constraints and height update algorithms based on dual-foot height differences are, respectively, designed. These algorithms aim to re-correct the pedestrian position information updated at zero velocity in both horizontal and vertical directions. The experimental results indicate that in a laboratory environment, the 3D positioning error is reduced by 93.9% compared to unconstrained conditions. Simultaneously, the proposed approach enhances the accuracy, continuity, and repeatability of the foot-mounted IMU positioning system without the need for additional power consumption.

## 1. Introduction

With the development of global navigation satellite systems (GNSS), they have become crucial infrastructures for spatiotemporal information systems, meeting the outdoor, wide-ranging, all-weather, and real-time high-precision navigation and positioning needs. However, satellite navigation signals are vulnerable, easily affected by obstructions such as buildings and dense vegetation, and often become ineffective indoors or in underground spaces [1,2]. To address this issue, various indoor positioning technologies based on radiofrequency stations, such as pseudo-satellites, ultra-wideband (UWB), and Bluetooth have been researched internationally [3,4,5]. Meanwhile, pedestrian navigation systems based on Micro-Electro-Mechanical System Inertial Measurement Units (MEMS-IMU), which do not rely on any external information, have gained widespread attention in the field of indoor positioning [6].

In recent years, scholars both domestically and internationally have conducted in-depth research on pedestrian navigation, focusing on foot-mounted, waist-mounted, and other distributed Inertial Measurement Unit (IMU) systems. Significant progress has been made in zero-velocity phase detection, human gait analysis, and compensation for inertial sensor errors. To enhance the accuracy of zero-velocity detection in mixed-motion modes, Yujie Sun [7], Mingkun Yang [8], and Seong Yun Cho [9] have proposed innovative zero-velocity interval (ZVI) detectors. To accommodate various motion patterns, Ni Zhu [10] proposed a machine learning model for detecting zero-velocity moments without any pre-classification step, named the Uniform AI Model for All pedestrian Motions (UMAM). Pedestrian gait detection helps improve the accuracy of foot-mounted pedestrian autonomous navigation. Zhihong Deng [11,12] has categorized common gaits into seven types and built a Bidirectional Long Short-Term Memory Recurrent Neural Network (BLSTM-RNN) as a gait classifier. By combining the Zero-Integration Heading Rate (ZIHR) method with a simplified Heuristic Drift Reduction (HDR) method, it reduces heading drift. Some scholars have also studied pedestrian gait detection and positioning with sensors installed at the waist and wrist. Nasim Hajati et al. [13] used an Inertial Measurement Unit (IMU) mounted on a belt, estimating the attitude through an unscented Kalman filter and ultimately calculating the position in three dimensions with the help of a step detection algorithm. Debjyoti Chowdhury et al. [14] developed a wearable device that can be fixed on the wrist, employing a simplified algorithm for human activity detection.

To enhance the robustness of the pedestrian autonomous navigation and localization system, Hongyu Zhao et al. [15] employed a threshold-based strategy to validate the detected zero-velocity update (ZUPT) periods. They proposed a quantitative metric for estimating the smoothness of the position data, with the advantage of achieving continuous error correction throughout the entire gait cycle. However, a limitation is its lack of adaptation to other walking speeds. Qiuying Wang et al. [16] introduced a rapid initial alignment algorithm for foot inertial/magnetic pedestrian localization based on an Adaptive Gradient Descent Algorithm (AGDA). This method considers the characteristics of gravity and the Earth’s magnetic field, measured by accelerometers and magnetometers when the pedestrian is stationary. Its advantage lies in the introduction of AGDA for quick initial alignment, but it must consider the impact of magnetic disturbances. Weixing Qian et al. [17] proposed a novel pedestrian navigation method that constructs an Adaptive Virtual Inertial Measurement Unit (VIMU) based on gait type classification. The advantage is the use of these models to address the problem of position estimation when the actual foot IMU is out of range. However, further improvement in accuracy is needed. Miaoxin Ji et al. [18] introduced a Zero-Velocity Update with Zero Position Difference (ZUPT-ZPD) combining foot and tibia kinematic information. To obtain accurate posture and position through the fusion of foot and tibia measurement information, they proposed an Improved Extended Kalman Particle Filter (EKPF) based on zero position difference to enhance localization accuracy. However, 3D pedestrian localization is not achieved. Tao Liu et al. [19] designed a personnel positioning system comprising a foot inertial module, a smartphone, and pre-deployed sparse QR code points. The system utilizes a newly developed data fusion algorithm for real-time and post-processing fusion of relative foot position and 3D QR code control point coordinate data. However, it requires pre-placement of QR code points. Wenchao Zhang et al. [20] proposed three improved constraint algorithms for detecting static stages, heading drift constraints, and height divergence constraints, respectively. Nevertheless, high-speed walking modes were not considered. Dongpeng Xie [21], based on a foot-carried pedestrian navigation system, employed an Error State Extended Kalman Filter (EKF) framework that integrates the GNSS position, zero-velocity state, barometric height, and other information. Its advantage lies in providing technical references for accurately and continuously obtaining common pedestrian position information. However, GNSS assistance is required. F. Seco et al. [22] estimated terrain slope and height changes during forward walking using IMU. The advantage is in checking whether the ramp is associated with existing ramps in the database, but it is not applicable to staircase scenarios.

Simultaneously, in the aforementioned studies, it was observed that pedestrian navigation systems based on foot-mounted inertial sensors have the drawback of decreasing position accuracy over long durations and distances due to error accumulation. Scholars both domestically and internationally have explored the use of pedestrian navigation systems based on dual inertial units to address this issue. Peter Händel et al. [23,24] proposed a method that fuses information from two navigation systems. The advantage lies in coupling two foot systems using the maximum separation distance constraint between the two systems, thereby improving the positioning performance. However, it is limited to the horizontal position correction. Prateek et al. [25] introduced a pedestrian navigation system based on the fusion of dual inertial systems. The advantage is in utilizing the maximum distance between the feet during normal walking to constrain the dual-system positioning results, achieving favorable outcomes, especially when the initial heading estimate is known. The team led by Xiaoji Niu at Wuhan University [26] discovered a reliable periodic equality constraint in pedestrian motion patterns. The advantage is an enhancement in the horizontal positioning performance of the dual-foot system. Exploiting the regularity in pedestrian bipedal motion, Wei Shi et al. [27], based on human kinematics, constructed an ellipsoid constraint model using the maximum stride length and foot lift height during walking. This approach improved pedestrian position estimation accuracy, although experiments across multiple walking speeds were not conducted. Ming Cheng et al. [28] proposed an improved method for dual-foot inertial/magnetometer pedestrian positioning based on adaptive inequality-constrained Kalman filtering. The advantage is the introduction of adaptive inequality constraints in the ZUPT Kalman filtering, reducing cumulative position errors, although the computational unit used has a larger volume. Sen Qiu et al. [29] established a low-cost body sensor network, leveraging a multi-sensor data fusion algorithm for gait analysis. The advantage is in using multiple sensors to achieve gait analysis, but the sensor quantity exceeds five. Renjie Wu et al. [30] addressed dual-foot inertial navigation systems (DF-INS) and proposed a Dual Trajectory Fusion (DTF) method. The advantage lies in merging left and right foot trajectories into a single center-of-mass trajectory using ZUPT clustering and fusion weight calculation. Shao Chen et al. [31] designed a dual MIMU single-board pedestrian inertial navigation system (PINS) and introduced a novel constraint method. The advantage is the formation of a constant three-dimensional (3D) position difference constraint based on the layout of two sensors on the circuit board, although other walking speeds were not considered. Min Su Lee et al. [32] developed an advanced human positioning algorithm using multiple wearable inertial sensors. The advantage is continuous fusion of position and velocity from a foot-based inertial measurement (PDR) system, leveraging known motion relationships between different body parts. However, a relatively large number of sensors were used.

Meanwhile, there are also some other correction methods for pedestrian positioning results based on inertial sensors. One common approach is to use indoor radio positioning base station information to correct the results of pedestrian inertial positioning. For instance, a combination of inertial sensors and UWB navigation methods can be employed, utilizing UWB ranging information to correct the inertial positioning results. The advantage of these methods is the ability to achieve higher precision in positioning results. However, a limitation is that it requires the pre-deployment of radio base stations.

In summary, in complex motion scenarios such as walking, running, ascending, and descending stairs, the pedestrian zero-velocity phase still cannot adaptively and accurately discriminate. The current pedestrian positioning method based on dual foot-mounted IMU has not defined the essence of dual-foot distance constraints. Addressing the above issues, this paper, based on the foot-mounted pedestrian navigation system and considering the application scenario of indoor navigation through extensive experimental analyses, makes contributions primarily in the following three aspects:

Firstly, an improved zero-velocity phase determination method based on generalized likelihood estimation is employed. This involves constructing a probability density function for inertial sensor data, adaptively adjusting the zero-velocity phase detection threshold based on the motion state and achieving a complete dual-foot zero-velocity updating and zero-height updating process.

Secondly, a horizontal position update algorithm based on dual-foot motion trajectory constraints is designed. In terms of horizontal localization, a method is proposed that uses the distance between the trajectory of one foot to the other foot as a constraint. The inequality Kalman filter method is utilized to correct the dual-system position.

Thirdly, in altitude localization, the height of the staircase between the dual feet is employed as observation data to further refine the altitude position results.

This paper is organized as follows: Section 2 analyzes the dual-foot gait characteristics and introduces the basic technical principles and mathematical models. Section 3 presents the dual-foot zero-velocity interval detection method. Section 4 designs the 3D pedestrian positioning scheme based on the dual foot-mounted IMU. Section 5 validates the effectiveness of the method by experimentally comparing the position accuracy before and after correction. Section 6 concludes the paper.

## 2. Basic Principles of Pedestrian Positioning

### 2.1. Gait Analysis for Dual Foot

**Figure 1:**
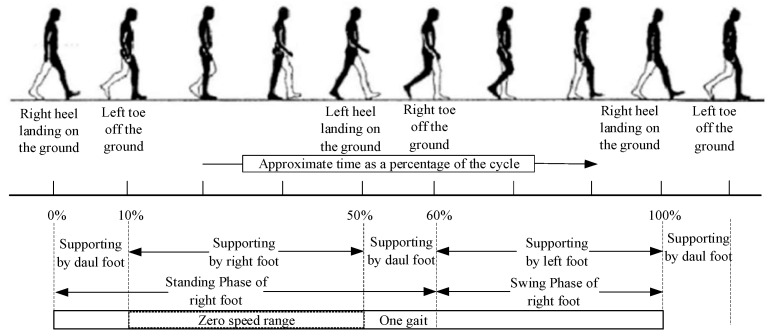
Schematic of the pedestrian gait period.

One gait is defined as the period between when a foot touches the ground and when the same foot touches the ground again [33]. In kinematics, the gait period of a pedestrian is divided into two phases: the standing phase and the swinging phase, where the standing phase, which refers to the process from heel strike to toe-off, is longer than the swinging phase and accounts for about 60% of the entire gait period [34]. The standing phase described in this paper differs from the definition of standing phase in kinematics; for example, the moment when the right foot is in full contact with the ground to the moment when the right heel is about to leave the ground, at which point the supporting foot is in full contact with the ground and has no relative displacement, is the standing phase referred to in this paper, and it is also referred to as the zero-velocity phase. Its percentage of the gait period varies depending on the gait speed, and within the zero-velocity phase, the current foot velocity should theoretically be zero. The pedestrian gait period is shown in Figure 1.

Based on foot-mounted indoor pedestrian inertial navigation systems, errors mainly arise from sensor measurements during zero-velocity phases, such as during the stationary phase. In this phase, the true estimated velocity should be 0, but the observed value calculated from sensor readings represents an error observation of the velocity during the zero-velocity phase.

### 2.2. Basic Zero-Velocity Correction Pedestrian Dead Reckoning Algorithm

**Figure 2:**
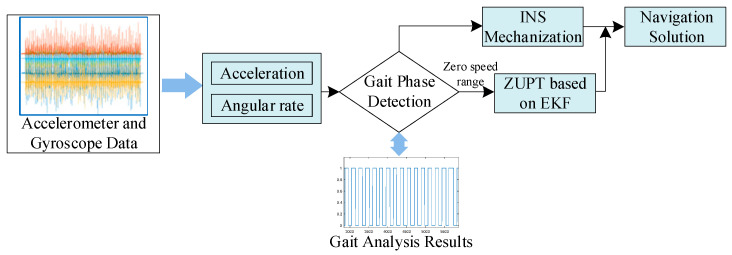
General framework of the basic zero-velocity correction pedestrian dead reckoning algorithm.

The Inertial Measurement Unit (IMU) can be installed on the body of pedestrians to acquire inertial data, measuring acceleration and angular rate information during the pedestrian’s motion. Through navigation algorithms that process and calculate the measured data, the IMU enables the recognition of gait patterns, as well as the positioning and navigation of pedestrians. As discussed in the previous section analyzing the bipedal gait of pedestrians, foot-mounted inertial sensors can also utilize the brief zero-velocity characteristics when the foot is fully grounded. This allows for partial error correction without the need for additional sensors. The overall framework of a basic zero-velocity-corrected pedestrian dead reckoning algorithm is illustrated in Figure 2. Commonly used zero-velocity correction estimation methods include Kalman filtering, quadratic curve fitting, curve fitting using the solution to the state equation, and maximum likelihood estimation.

The pedestrian navigation system based on foot-mounted IMU is nonlinear and can be corrected for navigation errors using the Extended Kalman Filter (EKF) [35]. Assuming the nonlinear system’s state equation and observation equation are

(1) $$\begin{array}{l} X_{k}=f(X_{k-1},k-1)+\Gamma (X_{k-1},k-1)W_{k-1}\\ Z_{k}=h(X_{k},k)+V_{k} \end{array}$$

In the equations, $X_{k}$ is the n-dimensional state sequence at time k, $Z_{k}$ is the m-dimensional observation sequence at time k, $W_{k-1}$ is the p-dimensional system process noise sequence at time k−1, and $V_{k}$ is the m-dimensional random observation noise sequence. $\Gamma (X_{k-1},k-1)$ is an n×p-dimensional matrix representing the system process noise input. The nonlinear function f is associated with the previous state vector and the current state vector, while the nonlinear function h is associated with the estimated state vector and the measurement vector. The state transition matrix $\Phi_{k,k-1}$ and the observation matrix $H_{k}$ are defined as

$$\Phi_{k,k-1}=\frac{\partial f}{\partial {\hat{X}}_{k-1}}=\frac{\partial f({\hat{X}}_{k-1},k-1)}{\partial X_{k-1}}\left|\begin{array}{c} {}_{X_{k-1}={\hat{X}}_{k-1}} \end{array}\right.,\;H_{k}=\frac{\partial h}{\partial X_{k}}\left|\begin{array}{c} {}_{{\hat{X}}_{k,k-1}} \end{array}\right.$$

(2) $${\hat{X}}_{k}={\hat{X}}_{k,k-1}+K_{k}[Z_{k}-h({\hat{X}}_{k,k-1},k)]$$

(3) $$K_{k}=P_{k,k-1}H_{k}^{T}{\left[H_{k}P_{k,k-1}H_{k}^{T}+R_{k}\right]}^{-1}$$

(4) $$P_{k,k-1}=\Phi_{k,k-1}P_{k-1}\Phi_{k,k-1}^{T}+\Gamma ({\hat{X}}_{k-1},k-1)Q_{k-1}\Gamma^{T}({\hat{X}}_{k-1},k-1)$$

(5) $$P_{k}=[I-K_{k}H_{k}]P_{k,k-1}$$

Formulas (2)–(5) represent the basic equations of discrete Kalman filtering, where $K_{k}$ is the Kalman gain, $P_{k,k-1}$ is the predicted associated estimation error covariance, $R_{k}$ is the variance matrix of $V_{k}$, $Q_{k-1}$ is the variance matrix of $W_{k-1}$, and $P_{k}$ is the updated associated estimation error covariance. By providing initial values for ${\hat{X}}_{0}$ and $P_{0}$, and based on the measurements $Z_{k}$ at time k, the estimated state ${\hat{X}}_{k}(k=1,2,\ldots )$ can be obtained.

This paper selects attitude error $\delta \varphi^{T}$, position error $\delta r^{T}$, and velocity error $\delta v^{T}$ as the state variables of the extended filter.

(6) $$\begin{array}{cc} X & ={\left[\begin{array}{ccc} \delta \varphi^{T} & \delta r^{T} & \delta v^{T} \end{array}\right]}^{T}\\ & ={\left[\begin{array}{ccccccccc} \delta \gamma & \delta \theta & \delta \psi & \delta r_{N} & \delta r_{E} & \delta r_{D} & \delta v_{N} & \delta v_{E} & \delta v_{D} \end{array}\right]}^{T} \end{array}$$

where δγ,δθ,δψ are attitude errors of the IMU mathematical platform, and $\delta r_{N},\delta r_{E},\delta r_{D}$ and $\delta v_{N},\delta v_{E},\delta v_{D}$ represent the position error and velocity error in the navigation coordinate system (i.e., north-east-down, NED). The linearized state transition model is given by

(7) $$X_{k}=\Phi_{k,k-1}X_{k-1}+w_{k-1}$$

The state transition matrix is $\Phi_{k,k-1}=\left[\begin{array}{ccc} I_{3\times 3} & 0_{3\times 3} & 0_{3\times 3}\\ 0_{3\times 3} & I_{3\times 3} & \Delta t\cdot I_{3\times 3}\\ -\Delta t\cdot S_{k} & 0_{3\times 3} & I_{3\times 3} \end{array}\right]$, where $S_{k}$ is represented as $S_{k}=\left[\begin{array}{ccc} 0 & -a_{zk}^{n} & a_{yk}^{n}\\ a_{zk}^{n} & 0 & -a_{xk}^{n}\\ -a_{yk}^{n} & a_{xk}^{n} & 0 \end{array}\right]$, $a_{xk}^{n},a_{yk}^{n},a_{zk}^{n}$ are the projection of the three-axis acceleration (x, y, z) in the navigation coordinate system at time k, Δt is the time interval between two sampling points, and $I_{3\times 3}$ denotes 3×3 the identity matrix.

Through the recursive process of extended Kalman filtering, estimates for attitude error, position error, and velocity error can be obtained. Feeding back the error estimates into the strapdown inertial navigation system allows for error compensation of various navigation parameters. At the current time, the velocity and position after error correction are given by

(8) $$\begin{array}{c} v_{k|k}=v_{k|k-1}-X_{k}(7:9)=v_{k|k-1}-\delta v_{k}\\ r_{k|k}=r_{k|k-1}-X_{k}(4:6)=r_{k|k-1}-\delta r_{k} \end{array}$$

where $v_{k|k-1}$ and $r_{k|k-1}$ are the uncorrected velocity and position at time k. The error correction of attitude angles is achieved by updating the attitude transformation matrix. The corrected attitude transformation matrix is given by

(9) $$C_{b_{k|k}}^{n}=g(C_{b_{k|k-1}}^{n},\delta \varphi_{k})=\frac{2I_{3\times 3}+\delta \Theta_{k}}{2I_{3\times 3}-\delta \Theta_{k}}\cdot C_{b_{k|k-1}}^{n}$$

where $C_{b_{k|k-1}}^{n}$ is the uncorrected attitude transformation matrix at time k, $\delta \varphi_{k}$ is the attitude error at time k, and $\delta \Theta_{k}$ is the skew-symmetric matrix of the attitude error at time k. The algorithm that utilizes the zero-velocity characteristics when the carrier is stationary for error correction is called the Zero-Velocity Update (ZUPT). Through the analysis of the pedestrian’s gait process, there is a brief standstill period during the gait cycle, known as the zero-velocity phase [36,37,38]. Due to factors such as noise and external disturbances, the actual velocity has some error, and it is not exactly zero during the standstill period. The velocity output during this period becomes the observation for the filter, as shown in Equation (10). This allows estimation of horizontal attitude errors, position errors, etc., which are then fed back into the navigation calculation system to obtain corrected navigation parameters.

(10) $$Z=v^{n}-0=\delta v^{n}$$

Correspondingly, the observation matrix is

(11) $$H=[\begin{array}{ccc} 0_{3\times 3} & 0_{3\times 3} & I_{3\times 3} \end{array}]$$

### 2.3. The Kalman Filter Based on Inequality Constraints

In the application of the discrete Kalman filter, the state variables are often limited by the environment and experimental conditions, and this limited information cannot be represented by a system model. If this information is ignored, the optimal solution in the actual situation will not be obtained, which will reduce the reliability of the estimation [39]. Therefore, the constraint equation is used to represent the constraint information; for example, inequality is used to express the relationship between the constraint information and the state variables, and then the optimal solution satisfying the constraint equation is calculated by combining with the Kalman filter [40], that is, the Kalman filter based on inequality constraints.

The optimal solution of the inequality-constrained Kalman filter can be set as

(12) $$\left\{\begin{array}{c} f(x_{k})=\underset{x}{\min}{\left({\hat{x}}_{k}-x_{k}\right)}^{T}W({\hat{x}}_{k}-x_{k}),\\ \varphi (x_{k})\leq d, \end{array}\right.$$

where $f(x_{k})$ is the objective function; W is a positive definite projective symmetry matrix, taken here as $W=P_{k}^{-1}$, where the covariance matrix of ${\hat{x}}_{k}$ is $P_{k}$; $\varphi (x_{k})$ is the mathematical equation of the limiting information; and d is the value of the constraints.

## 3. Improved Adaptive Zero-Velocity Phase Detection

The information acquisition module worn by the pedestrian’s left and right feet is mainly a six-degree-of-freedom inertial sensing unit composed of a three-axis gyroscope and a three-axis accelerometer, and its output model $y_{k}\in R^{6}$ is constructed to be denoted as

(13) $$y_{k}=\left[\begin{array}{c} y_{k}^{a}\\ y_{k}^{\omega } \end{array}\right]$$

where $y_{k}^{a}\in R^{3}$ is the matrix form of the acceleration ratio information at k moments and $y_{k}^{\omega }\in R^{3}$ is the matrix form of the angular velocity information at k moments. The main process of zero-velocity phase detection is to set N observations between n and n+(N−1) as time traversal phases to determine whether the foot-strapped sensor is moving or stationary under the condition that the original data sequence $z_{n}\overset{\Delta }{=}{\left\{y_{k}\right\}}_{k=n}^{n+N-1}$ is known. And the probability of the sensor remaining stationary is determined under the sensor non-stationary condition, while the probability of False Alarm is set to a very small value so that the probability of detecting it as a stationary event is maximized.

A mathematical model construction method is used to transform the detection of the zero-velocity phase into a binary hypothesis testing process by first setting up a detector chosen between hypothesis $H_{0}$ and hypothesis $H_{1}$, where $H_{0}$ denotes the non-zero-velocity phase of the MEMS-IMU and $H_{1}$ denotes the zero-velocity phase of the MEMS-IMU. The accuracy of the detector depends on the magnitude of the probability value of false alarms, which arise from the probability value $P_{FA}=P(H_{1}\left|H_{0}\right.)$ of judging that $H_{1}$ holds when hypothesis $H_{0}$ is true, and the probability value $P_{D}=P(H_{1}\left|H_{1}\right.)$ of judging accurately when hypothesis $H_{1}$ holds. The goal of judging two hypotheses at a given $P_{FA}$ by the Neyman–Pearson theorem is to maximize the value of $P_{D}$. Set $p(z_{n};H_{0})$ and $p(z_{n};H_{1})$ to represent the probability density functions of the two hypothesized observations, $P_{FA}=a$ is known and the conditions for judging $H_{1}$ to be true in order to maximize the value of $P_{D}$ are

(14) $$L(z_{n})=\frac{p(z_{n};H_{1})}{p(z_{n};H_{0})}>\gamma$$

The threshold γ in the above equation can be determined by the following equation:

(15) $$P_{FA}=\int_{\left\{z_{n}:L(z_{n})>\gamma \right\}}p(z_{n};H_{0})dz_{n}=\alpha$$

where the function $L(z_{n})$ is the likelihood ratio of traversing $z_{n}$, that is, the likelihood of hypothesis $H_{1}$ with respect to hypothesis $H_{0}$. Combined with the given mathematical model of the sensor, the generalized likelihood estimation decision condition is calculated by simplification as

(16) $$\frac{1}{N}\sum_{k\in \Omega_{n}}\left(\frac{1}{\sigma_{a}^{2}}{\left\|y_{k}^{a}-g\frac{{\bar{y}}_{n}^{a}}{\left\|{\bar{y}}_{n}^{a}\right\|}\right\|}^{2}+\frac{1}{\sigma_{\omega }^{2}}{\left\|y_{k}^{\omega }\right\|}^{2}\right)<\lambda$$

In this equation, the threshold for judgment is defined as λ=(-1/N)lnγ, where $\left\|{\bar{y}}_{n}^{a}\right\|$ represents the mean of acceleration divided by force output within the sliding window [41]. $\sigma_{a}^{2}$ and $\sigma_{\omega }^{2}$, respectively, denote the noise variance values for the accelerometer and gyroscope, while N stands for the current value of the sliding window.

This article introduces an enhanced threshold-adaptive zero-velocity interval detection algorithm which dynamically adjusts the judgment threshold λ in real-time by identifying different motion states. The primary challenge in adaptive threshold adjustment is how to recognize distinct motion states. In general, when a person is moving quickly, the peak magnitude of foot acceleration is noticeably greater than during slow movement, indicating higher fluctuations in inertial measurement data. These variations can be expressed through a series of mathematical features. Therefore, in motion state classification, real-time inertial measurement data are collected during motion. The average peak magnitude of acceleration within a 0.1-s sliding window (since the duration of foot contact during walking is relatively short, the window length should not be overly extended) is calculated. The current motion state is determined by analyzing this value.

After determining the motion state, the next challenge is how to select different zero-velocity interval detection thresholds for each motion state. Pedestrian motion, including normal walking, brisk walking, slow jogging, and fast running, is categorized into eight different speed levels. For each speed level, a total of 20 threshold calibration experiments are conducted. In these experiments, participants maintain a constant speed according to a predefined reference path. The goal is to find the detection threshold that minimizes trajectory error. This process is repeated 20 times for each speed level, resulting in statistical averages for the peak magnitude of acceleration (denoted as $\bar{{\left|a\right|}_{\max}}$) and the optimal detection threshold (denoted as $\lambda_{m}$), as shown in Table 1.

**Figure 3:**
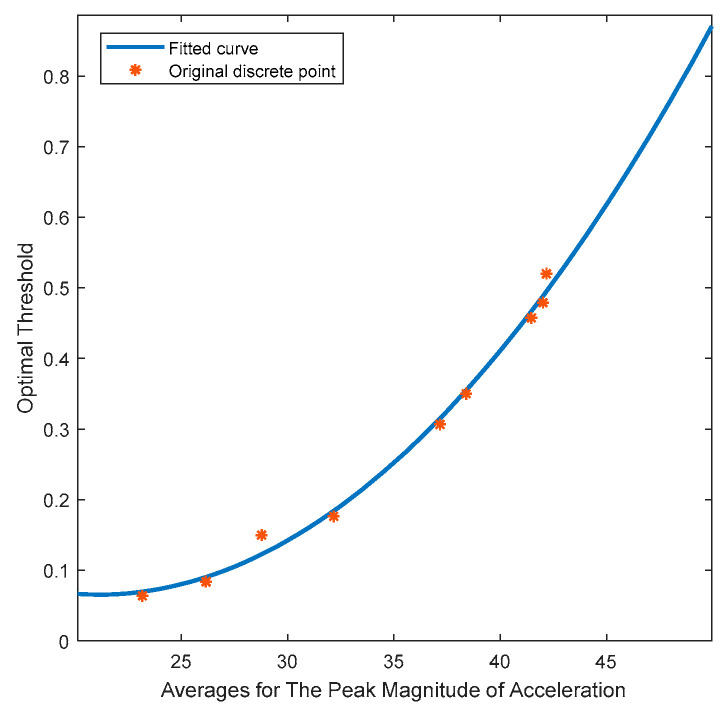
The function relationship between the optimal detection threshold and the average peak magnitude of acceleration.

Using polynomial fitting based on the data in the table, we can estimate the function relationship between the optimal detection threshold and the average peak magnitude of acceleration, as shown in Figure 3. The constructed function model is as follows:

(17) $$f(x)=9.6486e^{-4}x^{2}-0.0407x+0.4943$$

## 4. Pedestrian Autonomous 3D Positioning Algorithm Based on Dual-Foot Motion Characteristic Constraints

### 4.1. Analysis of Characteristics in Pedestrian Dual-Foot Motion Trajectories

Based on the analysis of dual-foot gait characteristics in Section 2.1, during various gait patterns in pedestrian locomotion, the left and right feet move alternately, with the distance between their trajectories staying within a certain range. In normal circumstances they do not intersect. From this pattern, we can deduce that in pedestrian bipedal navigation, the vertical distance between the right foot position and the left foot trajectory at any given moment remains within a maximum threshold, forming a constraint condition. This approach, distinct from constraints based on maximum step lengths or ellipsoidal constraints, is better suited for dynamic motions that involve significant variations in maximum step lengths, such as taking large strides or making turns.

**Figure 4:**
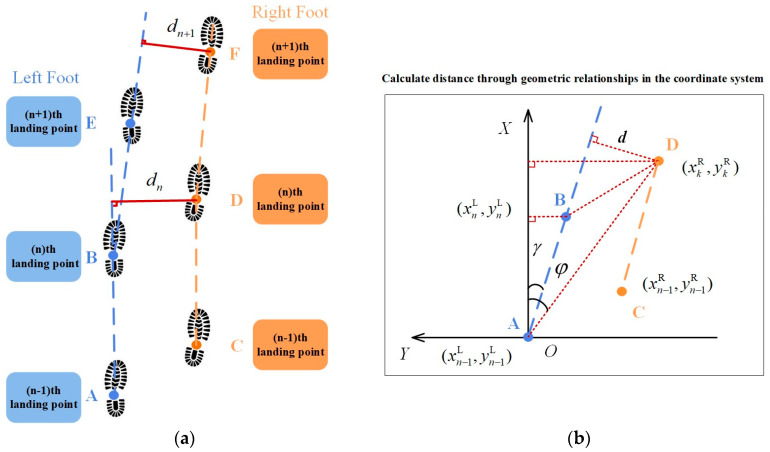
Horizontal distance constraints on dual-foot trajectories: (**a**) pedestrian dual-foot motion trajectories; (**b**) horizontal distance constraints.

In Figure 4a, a schematic representation of pedestrian dual-foot trajectories is presented. The dashed lines depict the projection of the trajectories onto the horizontal plane, and the black dots represent the landing points for the left and right feet. The solid lines connecting them provide an approximate representation of the movement trajectories between the two feet. To further analyze a stepping phase, the solid line connecting points A and B represents the trajectory of the left foot. Point A corresponds to the (n − 1)th landing point of the left foot, and point B represents the nth landing point of the left foot. Point C designates the (n − 1)th landing point of the right foot. During the phase when the left foot remains stationary and the right foot moves from its (n − 1)th to nth landing point (point D), the horizontal plane projection distance between the right foot and the AB trajectory is labeled as d, representing the vertical separation between the right foot’s position and the left foot’s trajectory at that specific moment, as illustrated in Figure 4b. In normal walking, d does not exceed a predetermined threshold denoted as η. Based on these motion characteristics, a secondary correction is applied from the horizontal direction to enhance the accuracy of the localization results.

The horizontal coordinates of the left and right feet at the (n − 1)th and nth landing points are defined as follows: The horizontal coordinate of point A is denoted as $p_{n-1}^{L}=(x_{n-1}^{L},y_{n-1}^{L})$, the horizontal coordinate of point B is denoted as $p_{n}^{L}=(x_{n}^{L},y_{n}^{L})$, and the horizontal coordinate of point C is denoted as $p_{n}^{R}=(x_{n-1}^{R},y_{n-1}^{R})$. The horizontal coordinate of the right foot at the kth moment is represented by point D, with its horizontal coordinate being $p_{k}^{R}=(x_{k}^{R},y_{k}^{R})$.

(18) $$\varphi =\arctan\left|\frac{x_{k}^{R}-x_{n-1}^{L}}{y_{k}^{R}-y_{n-1}^{L}}\right|$$

(19) $$\gamma =\arctan\left|\frac{x_{n}^{L}-x_{n-1}^{L}}{y_{n}^{L}-y_{n-1}^{L}}\right|$$

(20) $$d_{k}=\sqrt{(x_{k}^{R}-x_{n-1}^{L}{)}^{2}+{\left(y_{k}^{R}-y_{n-1}^{L}\right)}^{2}}\cdot \arcsin(\left|\varphi -\gamma \right|)$$

**Figure 5:**
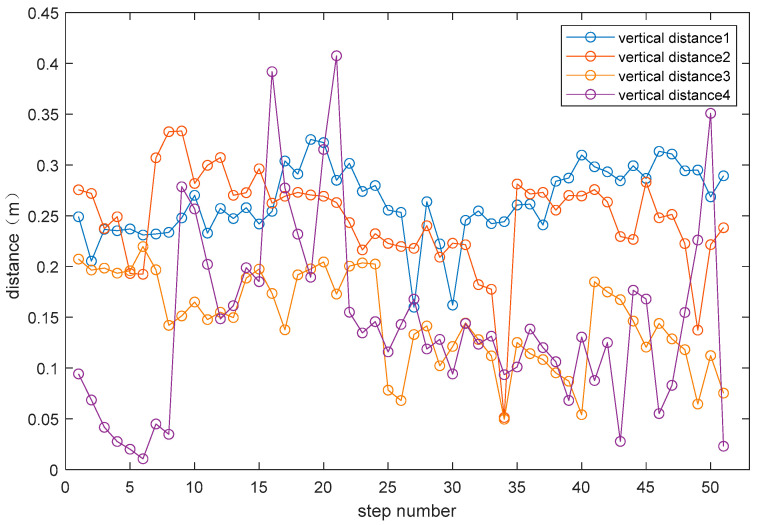
Statistical curve graph of the vertical distances between the right foot’s position during the standing phase and the trajectories of the left foot at different moments.

We utilized optical motion capture equipment to record the dual-foot motion trajectories of pedestrians and perform statistical analyses on the distance data between the right foot and left foot trajectories. This experiment was conducted using the Realis Optical Motion Capture System. Participants in the experiment attached optical markers (Markers) to the same location on the dorsum of both feet. Multiple motion capture cameras, positioned at various angles, continuously tracked these Marker points in real-time and transmitted their spatial coordinate data to a data processing workstation. We calculated the vertical distance between the right foot position during the standing phase of experimental subjects and the trajectory of the left foot at different moments, forming four groups of distance sequences. The curve graph is shown in Figure 5.

From Figure 5, it can be observed that the distance varied during pedestrian dual-foot motion, but it remained within a certain range. The maximum value did not exceed 0.407 m, and the minimum value did not fall below 0.011 m. The patterns of the distance sequences obtained from different experimental participants are similar; therefore, this maximum distance value can be used to establish an inequality constraint.

### 4.2. Horizontal Position Update Algorithm Based on Dual-Foot Motion Trajectory Constraints

In a pedestrian dual-foot navigation system, as analyzed in the preceding text, there is constraint information regarding the distance between bipedal trajectories. This relationship can be expressed using inequalities, and subsequently, the Kalman filter is constrained. By doing so, the optimal solution can be calculated, and the states estimated using this method better match the actual positioning results. The algorithm makes two main assumptions for its modeling. Firstly, it assumes that the inertial sensors worn on the feet are securely fixed and do not experience lateral displacement. Secondly, it assumes that the pedestrian’s movements include only conventional walking, slow running, fast running, jumping, and stair climbing modes. During these activities, the algorithm assumes that the pedestrian cannot subjectively control the placement of the feet and adheres to normal human activity patterns.

For two navigation systems, the true state of the ith navigation system at time k is represented by $x_{k}^{i}$ (including attitude, position, and velocity), and the estimated state is represented as ${\hat{x}}_{k}^{i}$, where $x_{k}^{i}\in {\mathbb{R}}^{n_{i}}$ and ${\hat{x}}_{k}^{i}\in {\mathbb{R}}^{n_{i}}$ are included. We define the joint state vector as follows:

(21) $$x_{k}\overset{\mathrm{def}}{=}{\left[\begin{array}{cc} {\left(x_{k}^{L}\right)}^{T} & {\left(x_{k}^{R}\right)}^{T} \end{array}\right]}^{T},{\hat{x}}_{k}\overset{\mathrm{def}}{=}{\left[\begin{array}{cc} {\left({\hat{x}}_{k}^{L}\right)}^{T} & {\left({\hat{x}}_{k}^{R}\right)}^{T} \end{array}\right]}^{T}$$

In the equation, ${\hat{x}}_{k}\in {\mathbb{R}}^{m}(n_{1}+n_{2}=m)$, ${\left(x_{k}^{L}\right)}^{T}={\left[\begin{array}{ccc} {\left(\delta \varphi_{k}^{L}\right)}^{T} & {\left(\delta r_{k}^{L}\right)}^{T} & {\left(\delta v_{k}^{L}\right)}^{T} \end{array}\right]}^{T}$, ${\left(x_{k}^{R}\right)}^{T}={\left[\begin{array}{ccc} {\left(\delta \varphi_{k}^{R}\right)}^{T} & {\left(\delta r_{k}^{R}\right)}^{T} & {\left(\delta v_{k}^{R}\right)}^{T} \end{array}\right]}^{T}$.

When pedestrians move on the same floor, at time k, the vertical distance between the time trajectories of the right foot and left foot front and back positions, calculated as $d_{k}$, can be described in relation to a threshold:

(22) $$\sqrt{{\left\|R_{x}\cdot {\hat{x}}_{k}\right\|}^{2}+{\left\|R_{y}\cdot {\hat{x}}_{k}\right\|}^{2}}\cdot \arcsin(\arctan\left|\frac{R_{x}\cdot {\hat{x}}_{k}-x_{n-1}^{L}}{R_{y}\cdot {\hat{x}}_{k}-y_{n-1}^{L}}\right|-\arctan\left|\frac{x_{n}^{L}-x_{n-1}^{L}}{y_{n}^{L}-y_{n-1}^{L}}\right|)\leq \eta$$

In the equation, $R_{x}=\left[\begin{array}{ccc} 0_{1\times 12} & \begin{array}{cc} \begin{array}{cc} 1 & 0 \end{array} & 0 \end{array} & 0_{1\times 3} \end{array}\right]$, $R_{y}=\left[\begin{array}{ccc} 0_{1\times 12} & \begin{array}{cc} \begin{array}{cc} 0 & 1 \end{array} & 0 \end{array} & 0_{1\times 3} \end{array}\right]$.

The problem can be solved by the local minimum point of quadratic programming and the maximum likelihood method, as shown by (23). The state vector for navigation estimation is constrained within a reasonable subspace, calculating a state estimation vector with higher precision that fulfills the inequality constraint:

(23) $$\left\{\begin{array}{c} p({\hat{x}}_{k})\overset{\mathrm{def}}{=}\arg_{x}\min{\left({\hat{x}}_{k}-x_{k}\right)}^{T}P_{k}^{-1}({\hat{x}}_{k}-x_{k})\\ d_{k}\leq \eta \end{array}\right.$$

In the equation, $x_{k}$ denotes the estimated results of unconstrained Kalman filter state variables; $P_{k}^{}$ stands for the unconstrained Kalman filter covariance matrix; and ${\hat{x}}_{k}$ signifies the estimated results of constrained Kalman filter state variables.

### 4.3. Height Update Algorithm Based on the Height Difference between Both Feet

**Figure 6:**
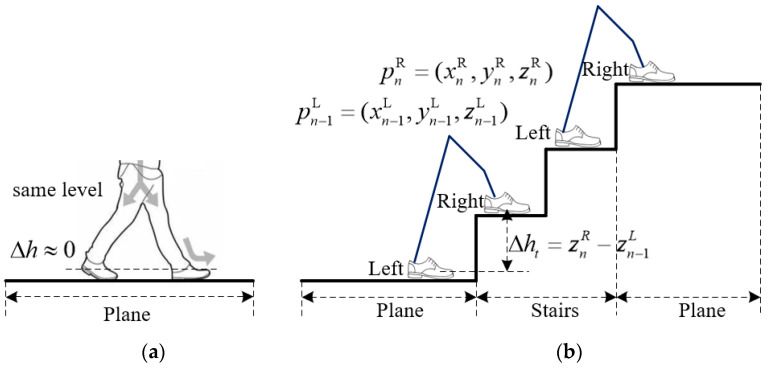
Schematic diagram of pedestrian height update in flat and staircase scenarios: (**a**) flat scene; (**b**) stairs scene (ascending and descending).

In the process of bipedal locomotion by pedestrians on the same plane, the theoretical value of the height difference between the two feet, calculated after each step is taken with the left and right feet, should be 0, as shown in Figure 6a. Therefore, inspired by the zero-velocity update algorithm, the Dual-foot Zero Height Update (DZHU) algorithm is proposed. It updates the pedestrian’s height coordinates in the zero-velocity phase to obtain a more accurate height estimation.

The left and right foot initial coordinates are defined as $p_{n-1}^{L}=(x_{n-1}^{L},y_{n-1}^{L},z_{n-1}^{L})$ and $p_{n-1}^{R}=(x_{n-1}^{R},y_{n-1}^{R},z_{n-1}^{R})$, respectively. After the right foot takes a step, the coordinates become $p_{n}^{R}=(x_{n}^{R},y_{n}^{R},z_{n}^{R})$. The calculated height difference between both feet is represented as $\Delta h_{t}=z_{n}^{R}-z_{n-1}^{L}$. If the current value of $\Delta h_{t}$ is less than a pre-defined threshold, it is considered that the pedestrian is walking on a flat surface, and the height difference between the feet remains unchanged. $\Delta h_{t}$ represents the observed error in the height coordinates at time t in the zero-velocity phase. Otherwise, the pedestrian’s height has truly changed, possibly due to activities such as ascending or descending stairs, and further analysis and verification are required, as explained below.

In the normal process of ascending and descending stairs, the height difference between the dual feet varies, rendering the dual-foot zero height update algorithm no longer applicable. In such situations, the height difference between the two feet is fixed at the height of m(m=1 or 2 or 3,m<4) steps, as shown in Figure 6b. Through research and analysis, we propose a height estimation algorithm that utilizes the height between the two feet as an observation. The prerequisite for applying this algorithm is that the pedestrian is not walking on an inclined floor or ramp and that the height of each step of indoor building stairs is nearly the same, meeting relevant industry standards. The algorithm’s modeling is based on specific assumptions and is only applicable for correcting height positioning results during extended stair descent. It cannot achieve height positioning correction in scenarios such as long slopes or elevators.

Determining m is pivotal in this context. Initially, the known height of an individual staircase step is defined as $\Delta h_{0}$. By assessing the height difference $\Delta h_{t}$ between the two feet within various threshold ranges, an estimation of the number of steps m between the left and right feet is achieved. This process aims to obtain the observed error $m*\Delta h_{0}$ in the height coordinates at time t. Subsequently, these observed errors are integrated into the observation vector Z, resulting in the formulation depicted in Equation (24).

(24) $$Z=\left\{\begin{array}{cc} \left[\Delta h_{t}\;\delta v^{n}\right] & \left|\Delta h_{t}\right|<h_{1}\\ \left[(\Delta h_{t}-1*\Delta h_{0})\delta v^{n}\right] & \;\;\;\;\;h_{1}\leq \left|\Delta h_{t}\right|<h_{2}\\ \left[(\Delta h_{t}-2*\Delta h_{0})\delta v^{n}\right] & \;\;\;\;\;h_{2}\leq \left|\Delta h_{t}\right|<h_{3}\\ \left[(\Delta h_{t}-3*\Delta h_{0})\delta v^{n}\right] & \left|\Delta h_{t}\right|\geq h_{3} \end{array}\right.$$

In the equation, $\delta v^{n}$ represents the velocity error vector. In the experiments described in this paper, $h_{1}=0.1\;m$ is defined, $h_{2}=0.2\;m$ is set to a value between the height of one and two steps, and $h_{3}=0.4$ is set to a value between the height of two and three steps.

## 5. Pedestrian Navigation System Prototype Construction and Experimental Validation

### 5.1. Construction of a Pedestrian Navigation System

**Figure 7:**
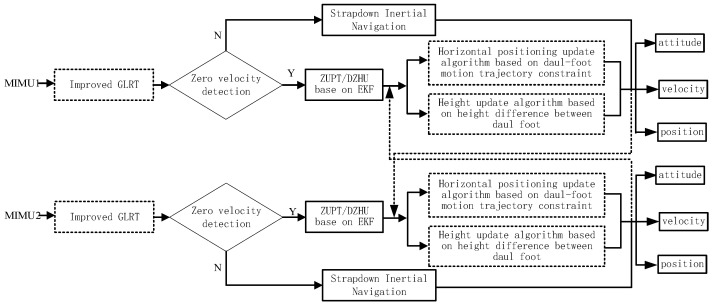
The schematic diagram of the improved method for indoor 3D pedestrian positioning based on dual foot-mounted IMU system.

Figure 7 outlines the workflow from the raw input data from the bipedal system to the output of three-dimensional positioning results. To establish a dual foot-mounted pedestrian navigation system, IMUs are worn on the left and right feet of the human body, collecting foot angular velocity and acceleration data at the same frequency. Utilizing an improved zero-velocity detection method based on a generalized likelihood ratio, it accurately determines whether the pedestrian is in a static phase. If the detection output is false, each system calculates attitude, velocity, and position information through loosely coupled integration. If the output is true, the zero-velocity update method and the dual-foot zero height update method are employed to correct the velocity and height information. Furthermore, based on constraints from the characteristics of bipedal motion, an inequality-constrained Kalman filter is constructed. This filter performs a secondary correction of the pedestrian’s horizontal position after zero-velocity correction. Using the prior knowledge of the step height between the feet as an observation, it updates the pedestrian’s height coordinates during the zero-velocity phase to obtain a more robust three-dimensional positioning result.

**Figure 8:**
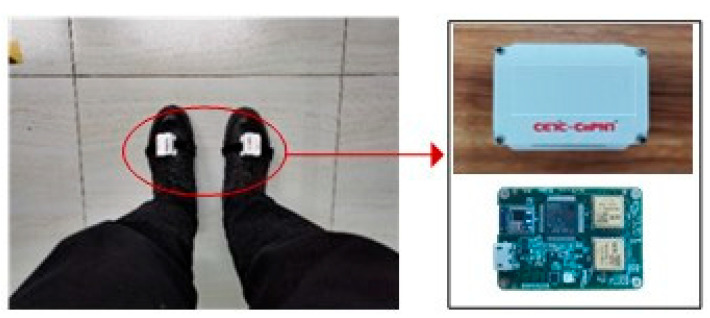
Physical image of the wearable dual-foot pedestrian navigation microsystem.

To validate the feasibility of the dual-foot pedestrian navigation system and its positioning method proposed in this paper, each foot of the pedestrian is equipped with a set of wearable positioning terminals with the same accuracy, as illustrated in Figure 8. The hardware of the terminal mainly includes a three-axis accelerometer, a three-axis gyroscope chip, a processor, a Bluetooth module, a battery, etc., with detailed performance parameters outlined in Table 2. The two terminals can achieve real-time communication through the Bluetooth module and are also capable of receiving and collecting raw sensor data through serial communication.

### 5.2. Experimental Validation

**Figure 9:**
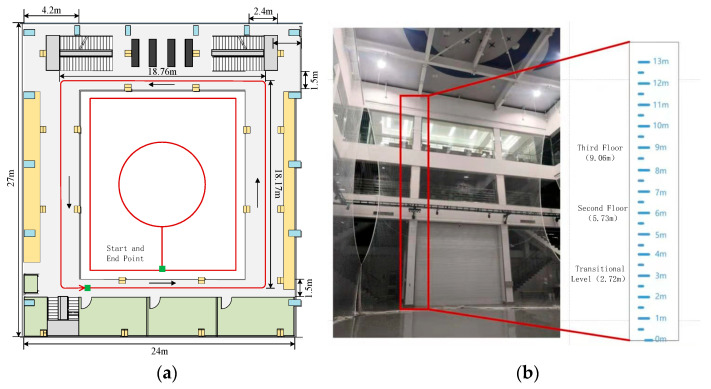
Experimental testing environment and paths: (**a**) pedestrian horizontal positioning experiment with a predefined route; (**b**) pedestrian vertical positioning experiment with a predefined route.

To validate the performance of the algorithm in both horizontal and vertical directions and to facilitate the accuracy assessment, closed walking routes were selected in both horizontal and vertical directions. The positioning error was determined by comparing the deviation between the final calculated position and the starting point. The experimental site was the three-story building of the Artificial Intelligence Navigation Test Field. The paths are of various types such as rectangles, straight lines, circles, etc. Adhesive tape was pre-applied to the floor to facilitate the experimenters in walking along the routes. Horizontal paths were set up in the first-floor hall and the second-floor rectangular corridor, while the vertical positioning experiments were conducted on the staircase from the first to the third floor. There are no slopes, damaged steps, etc. The spacious staircase environment also facilitates experimenters in achieving different walking speeds when going up and down the stairs. The specific environment and path planning are illustrated in Figure 9.

**Figure 10:**
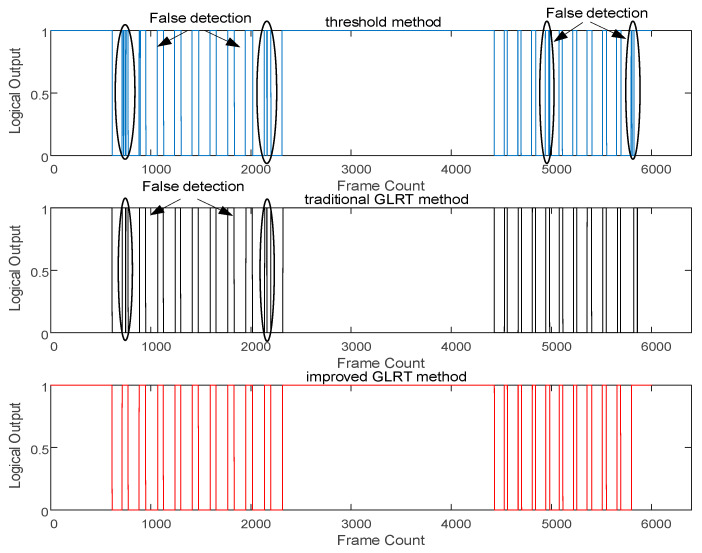
The zero-velocity detection results for walking and fast walking motion states for the three methods.

**Figure 11:**
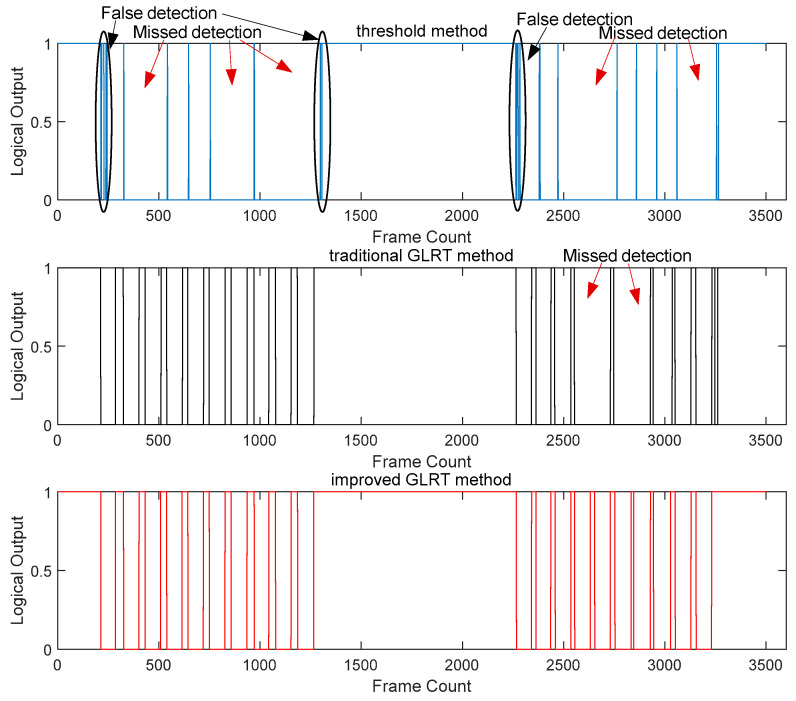
The zero-velocity detection results of the three methods under slow jogging and fast running motion states.

Experiment 1: To validate the dynamic adjustability of the improved adaptive zero-velocity phase detection method, the same individual collected experimental data in four different motion states—walking, brisk walking, slow running, and fast running—along the same path. The actual step counts were recorded for each state, and the results were compared with the classical threshold method and the traditional GLRT method with a fixed threshold. The comparative results are illustrated in Figure 10 and Figure 11.

Figure 10 presents the detection results of the three methods in walking and brisk walking states. The blue line, black line, and red line represent the results of the classical threshold method, the GLRT method with a fixed threshold, and the proposed method in this paper, respectively. It can be observed that the classical threshold method and the GLRT method with a fixed threshold have instances of missed detection. The zero-velocity phase detection accuracies for the three methods are 80%, 90%, and 100%, respectively. Figure 11 illustrates the detection results in the slow running and fast running states, with the zero-velocity phase detection accuracies for the three methods being 65%, 85%, and 100%, respectively. The classical threshold-based zero-velocity interval detection method exhibited severe missed detections and frequent false alarms. Additionally, in the fast running state, the fixed threshold GLRT method showed an increased rate of missed detections, indicating that these two methods are no longer suitable for fast running motion states.

Due to the use of fixed detection thresholds in the classical threshold method and the GLRT method with a fixed threshold, they often achieved satisfactory results only in specific motion states. The method proposed in this paper adaptively adjusts the detection threshold according to the motion state, ensuring accurate zero-velocity phase detection in all three states.

Experiment 2: This experiment involved two sets of paths. In one set, the experimental subjects walked at a normal pace from the starting point, circled around a rectangular path, and returned to the original point. In the other set, the experimental subjects walked at a normal pace from the starting point, followed a path that included both rectangular and circular sections, and returned to the original point. After the devices were powered on, the pedestrians remained stationary for the first 10 s, and the sampling frequency was set to 100 Hz. During the walking process, it was assumed that the wearable terminal was rigidly attached to the foot and that no sliding occurred.

**Figure 12:**
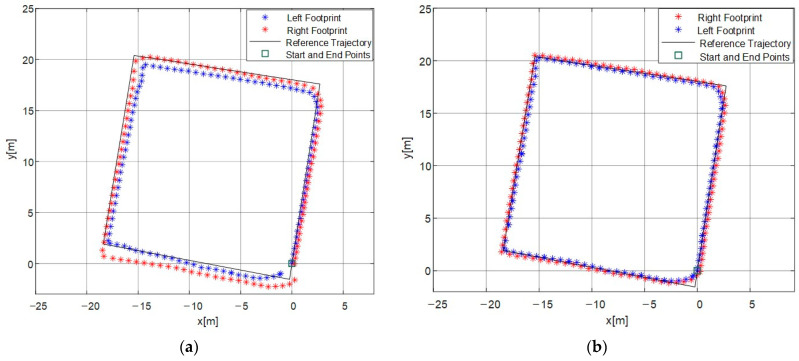
Positions of the left and right foot landing points before and after constraint: (**a**) unconstrained positions of the left and right foot landing points before correction; (**b**) positions of the left and right foot landing points after inequality-constrained Kalman filtering.

By comparing the positions of the left and right foot landing points before and after constraint, firstly, the unconstrained positions of the dual-foot landing points are shown in Figure 12a. From the figure, it can be analyzed that the distance between the trajectories of the right and left foot accumulates and gradually becomes larger over time, especially increasing at turning points. The distance is relatively stable during straight walking. Errors in heading angles lead to irregular trajectory shapes, and the average error for the final return of the left and right feet to the original point is 1.548 m. The trajectories formed by the left and right foot landing points show a trend of separation, with the distance between the trajectories gradually increasing to a degree that deviates from the typical human motion pattern. After applying constraints based on the characteristics of dual-foot motion, the landing points are shown in Figure 12b. Firstly, it can be observed that the trajectories are more regular after constraint, aligning better with the predefined trajectory. The correction effect of the landing points is significant, especially at turning points. The average error for the final return of the left and right feet to the original point is 0.498 m after constraint, resulting in a 67.8% reduction in positioning error compared to the unconstrained case. Moreover, there is a regular alternation between the left and right foot landing points, conforming to the typical human walking pattern. It can be observed that the constrained trajectory results exhibit higher continuity and reliability.

**Figure 13:**
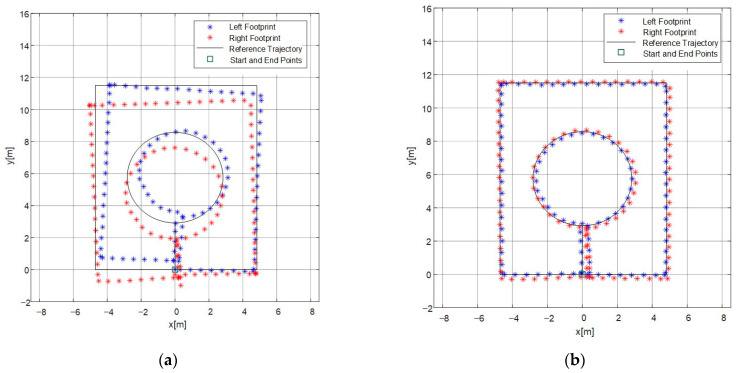
Positions of the left and right foot landing points before and after constraint in a complex path: (**a**) unconstrained positions of the left and right foot landing points before correction; (**b**) positions of the left and right foot landing points after inequality-constrained Kalman filtering.

Using the same method, a second set of experiments was conducted to verify the effectiveness of the algorithm in more complex paths. Since the distance between the trajectories of the right and left foot varies, the circular path poses a challenge for this algorithm. The trajectory of the rectangle is more rectangular, and when examining the same straight path traversed in different instances, the position results calculated before and after are close. This indicates that the method proposed in this paper improves the repeatability of pedestrian autonomous positioning. From the Figure 13, it can be analyzed that the constrained dual-foot trajectory points better align with the circular reference path. The average positioning error for the left and right feet is 0.423 m after constraint, which is a 49.5% reduction compared to the unconstrained case with an error of 0.838 m. This indicates that the algorithm based on dual-foot motion characteristics in this paper improves the positioning accuracy in the horizontal direction. And there is no additional radio positioning sensor module, maintaining the low power consumption characteristics of this solution.

Experiment 3: Height positioning experiments were conducted on stairs, both ascending and descending, in a three-story stairwell. Participants powered up the devices and collected experimental data by walking from the first floor to the third floor and vice versa. Using a laser rangefinder, measurements were taken to determine that the total height of the three floors was 9.06 m, with each step having a height of 0.16 m. During this process, the left and right feet were set to alternate between ascending and descending stairs, ensuring that the real vertical distance between the feet corresponds to the height of one step. The horizontal distance between the feet was not considered throughout the process, and it was assumed that the devices were worn on shoes without experiencing relative displacement.

**Figure 14:**
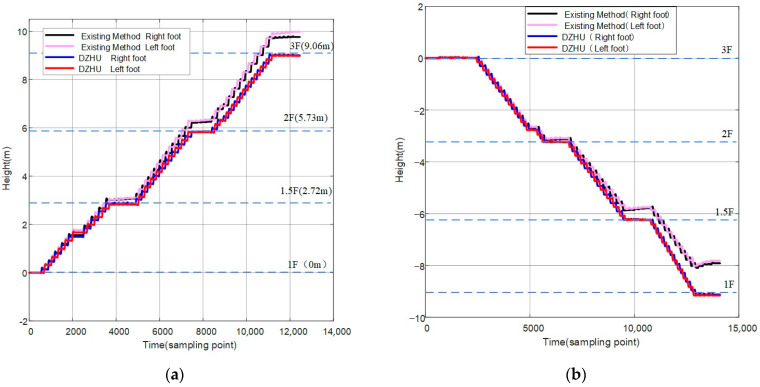
Curve charts of left and right foot height positioning before and after constraints: (**a**) comparison chart of bilateral height positioning results before and after constraints while ascending stairs; (**b**) comparison chart of bilateral height positioning results before and after constraints while descending stairs.

The experimental results are shown in Figure 14, where the horizontal axis represents the frame number of the raw data, and the vertical axis represents the height value. Analyzing the graph, it can be observed that during the process of ascending and descending stairs, the existing basic algorithm has multiple sources of acceleration errors in the vertical direction. This leads to a significant divergence in the results of the vertical height positioning of the feet over time, gradually losing the ability to determine the floor. The figure shows that the one with a larger height positioning error is 1.24 m. At the same time, it can be analyzed that the height difference between the two feet is gradually increasing, which is inconsistent with the assumed condition in the actual process that the height difference between the two feet remains within a certain range.

The height positioning results based on the Dual-Foot Height Difference Updating Algorithm (DZHU) gradually approach reasonable values. By utilizing the height difference between both feet, it restricts the tendency of height divergence, providing a more complete display of the pedestrian’s stair ascent process. The height positioning errors using this method for ascending and descending stairs are 0.07 m and 0.08 m, respectively. This represents a significant reduction in error compared to the existing baseline algorithm, with a decrease of 91.3% and 93.2% for the ascending and descending stairs, respectively. This indicates an improvement in positioning accuracy in the vertical direction through the height updating algorithm (DZHU). Similarly, this algorithm has improved the reliability and repeatability of the height measurement method based on low-cost inertial sensors. This is beneficial for the practical application of low-cost inertial sensor modules in engineering.

Experiment 4: Walking indoors from the starting point, climbing one set of stairs to the second floor, and then descending the other set of stairs back to the starting point. During the straight path, the participants walked at different speeds, including fast walking, jogging, and running. The subjects wore the dual-foot positioning terminal, and the experiment followed the ‘complex’ path, recording data along the way.

**Figure 15:**
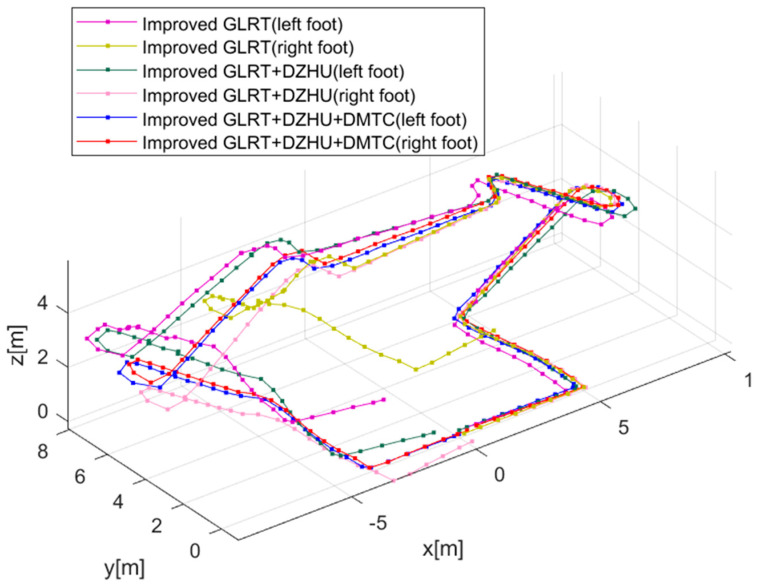
The trajectory comparison of the three methods for solving the 3D positioning of the pedestrian dual-foot system.

The data processing was carried out using three methods: Improve GLRT, Improve GLRT + DZHU, and Improve GLRT + DZHU + DMTC, as shown in Figure 15. The average of the 3D positioning errors of the left and right feet was taken as the position error result. Due to the drawback of error accumulation in low-cost inertial sensors and the lack of further correction of measurement results in both horizontal and vertical directions, using the Improve GLRT method alone resulted in a positioning error of 3.435 m, which is not conducive to position measurements in practical complex environments. By applying the Improve GLRT + DZHU method, the divergence in the vertical direction was constrained, leading to a positioning error of 0.679, significantly improving the positioning accuracy. The position error using Improve GLRT + DZHU is 0.679 m, and the result obtained using Improve GLRT + DZHU + DMTC is better than the other two methods, with a positioning error of 0.209 m. The accuracy is improved by 93.9% and 69.2%, respectively. The proposed method not only enhances the positioning accuracy of the system in practical complex environments but also improves the system’s continuity, reliability, and repeatability.

## 6. Conclusions

This article proposes an improved autonomous 3D positioning algorithm for pedestrians based on data from micro-inertial sensors such as gyroscopes and accelerometers worn on the pedestrian’s feet. The algorithm utilizes constraints based on the motion characteristics of both feet to correct the 3D positioning of pedestrians indoors. To achieve this, a pedestrian 3D positioning system was constructed using dual foot-mounted IMUs. The proposed algorithm incorporates an improved adaptive zero-velocity interval detection method, along with a horizontal positioning update algorithm based on constraints from the motion trajectories of both feet and a height update algorithm based on the height difference between both feet. This approach is better suited for three-dimensional positioning scenarios involving variations in walking speed and navigating stairs. The results of this study demonstrate that our autonomous pedestrian positioning trajectory correction method exhibits excellent measurement positioning performance, reliability, and repeatability. Additionally, the designed dual foot-mounted IMU module is compact, requires low power, and is easy to wear, making it suitable for prolonged use. The main limitation of our work is the need for further research into the integration and fusion of multiple types of miniature sensors. In the future, we aim to develop a more cost-effective, low-power, and comfortable-to-wear positioning microsystem.

## Figures and Tables

**Table 1:** Relationship between average peak magnitude of acceleration and optimal detection threshold.

Test	Normal Walking		Brisk Walking		Slow Jogging		Fast Running		
average peak magnitude of acceleration	23.16	26.15	28.77	32.16	37.16	38.40	41.46	42.0	42.17
optimal detection threshold	0.0638	0.0839	0.150	0.176	0.307	0.350	0.457	0.479	0.520

**Table 2:** The main characteristics of wearable positioning terminals.

Parameter	Gyroscope inside the Wearable Terminal	Accelerometer Inside the Wearable Terminal
Update Rate	100 Hz	100 Hz
Standard full range	450°/s	20 g
In-run bias stability	18°/h	15 μg
Noise density	0.03${}^{\circ}/s/\sqrt{\mathrm{Hz}}$	60 $\mu g/\sqrt{\mathrm{Hz}}$

## Data Availability

Data are contained within the article.

## References

[1] Xin L., Peng Z., Jiming G. (2017). A New Method for Single-Epoch Ambiguity Resolution with Indoor Pseudolite Positioning. Sensors.

[2] Seguel F., Palacios-Játiva P., Azurdia-Meza C.A., Krommenacker N., Charpentier P., Soto I. (2022). Underground Mine Positioning: A Review. IEEE Sens. J..

[3] Zhang B., Wang Q., Xia W., Sun Y., Wang J. (2023). Pseudolite Multipath Estimation Adaptive Mitigation of Vector Tracking Based on Ref-MEDLL. Remote Sens..

[4] Guo Y., Li W., Yang G., Jiao Z., Yan J. (2022). Combining Dilution of Precision and Kalman Filtering for UWB Positioning in a Narrow Space. Remote Sens..

[5] Zirari S., Canalda P., Spies F. WiFi GPS based combined positioning algorithm. Proceedings of the 2010 IEEE International Conference on Wireless Communications, Networking and Information Security.

[6] Xu Y., Li G., Li Z., Yu H., Cui J., Wang J., Chen Y. (2022). Smartphone-Based Unconstrained Step Detection Fusing a Variable Sliding Window and an Adaptive Threshold. Remote Sens..

[7] Sun Y., Xu X., Tian X., Zhou L., Li Y. (2021). An Adaptive Zero-Velocity Interval Detector Using Instep-Mounted Inertial Measurement Unit. IEEE Trans. Instrum. Meas..

[8] Yang M., Zhu R., Xiao Z., Yan B. (2022). Symmetrical-Net: Adaptive Zero Velocity Detection for ZUPT-Aided Pedestrian Navigation System. IEEE Sens. J..

[9] Cho S.Y., Lee J.H., Park C.G. (2022). A Zero-Velocity Detection Algorithm Robust to Various Gait Types for Pedestrian Inertial Navigation. IEEE Sens. J..

[10] Kone Y., Zhu N., Renaudin V. (2022). Zero Velocity Detection Without Motion Pre-Classification: Uniform AI Model for All Pedestrian Motions (UMAM). IEEE Sens. J..

[11] Deng Z., Wang P., Yan D., Shang K. (2020). Foot-Mounted Pedestrian Navigation Method Based on Gait Classification for Three-Dimensional Positioning. IEEE Sens. J..

[12] Deng Z., Wang P., Liu T., Cao Y., Wang B. (2019). Foot-mounted pedestrian navigation algorithm based on BOR/MINS integrated framework. IEEE Trans. Ind. Electron..

[13] Hajati N., Rezaeizadeh A. (2021). A Wearable Pedestrian Localization and Gait Identification System Using Kalman Filtered Inertial Data. IEEE Trans. Instrum. Meas..

[14] Chowdhury D., Chattopadhyay M. (2020). Development of a Low-Power Microcontroller-Based Wrist-Worn Device With Resource-Constrained Activity Detection Algorithm. IEEE Trans. Instrum. Meas..

[15] Zhao H., Wang Z., Gao Q., Hassan M.M., Alelaiwi A. (2015). Smooth estimation of human foot motion for zero-velocity-update-aided inertial pedestrian navigation system. Sens. Rev..

[16] Wang Q., Yin J., Noureldin A., Iqbal U. (2018). Research on an Improved Method for Foot-Mounted Inertial/Magnetometer Pedestrian-Positioning Based on the Adaptive Gradient Descent Algorithm. Sensors.

[17] Qian W., Zhu Y., Jin Y., Yang J., Qi P., Wang Y., Ma Y., Ji H. (2021). A Pedestrian Navigation Method Based on Construction of Adapted Virtual Inertial Measurement Unit Assisted by Gait Type Classification. IEEE Sens. J..

[18] Ji M., Xu X., Li Z., Wang J., Liu J. (2021). A Zero-Position-Difference ZUPT Method for Foot-Shank-Mounted Pedestrian Inertial Navigation Systems. IEEE Sens. J..

[19] Liu T., Kuang J., Ge W., Zhang P., Niu X. (2021). A Simple Positioning System for Large-Scale Indoor Patrol Inspection Using Foot-Mounted INS, QR Code Control Points, and Smartphone. IEEE Sens. J..

[20] Zhang W., Wei D., Yuan H. (2020). The Improved Constraint Methods for Foot-Mounted PDR System. IEEE Access.

[21] Xie D., Jiang J., Wu J., Yan P., Tang Y., Zhang C., Liu J. (2022). A Robust GNSS/PDR Integration Scheme with GRU-Based Zero-Velocity Detection for Mass-Pedestrians. Remote Sens..

[22] Jiménez A., Seco F., Zampella F., Prieto J., Guevara J. Ramp Detection with a Foot-Mounted IMU for a Drift-Free Pedestrian Position Estimation. Proceedings of the International Conference on Indoor Positioning and Indoor Navigation.

[23] Girisha R., Prateek G.V., Hari K.V.S., Handel P. Fusing the navigation information of dual foot-mounted zero-velocity-update-aided inertial navigation systems. Proceedings of the 2014 International Conference on Signal Processing and Communications (SPCOM).

[24] Skog I., Nilsson J.-O., Zachariah D., Handel P. Fusing the information from two navigation systems using an upper bound on their maximum spatial separation. Proceedings of the 2012 International Conference on Indoor Positioning and Indoor Navigation (IPIN).

[25] Prateek G.V., Girisha R., Hari K.V.S., Handel P. Data Fusion of Dual Foot-Mounted INS to Reduce the Systematic Heading Drift. Proceedings of the 2013 4th International Conference on Intelligent Systems, Modelling and Simulation.

[26] Niu X., Li Y., Kuang J., Zhang P. (2019). Data Fusion of Dual Foot-Mounted IMU for Pedestrian Navigation. IEEE Sens. J..

[27] Shi W., Wang Y., Wu Y. (2017). Dual MIMU Pedestrian Navigation by Inequality Constraint Kalman Filtering. Sensors.

[28] Wang Q., Cheng M., Noureldin A., Guo Z. (2019). Research on the improved method for dual foot-mounted Inertial/Magnetometer pedestrian positioning based on adaptive inequality constraints Kalman Filter algorithm. Measurement.

[29] Qiu S., Liu L., Zhao H., Wang Z., Jiang Y. (2018). MEMS Inertial Sensors Based Gait Analysis for Rehabilitation Assessment via Multi-Sensor Fusion. Micromachines.

[30] Wu R., Lee B.G., Pike M., Zhu L., Chai X., Huang L., Wu X. (2022). IOAM: A Novel Sensor Fusion-Based Wearable for Localization and Mapping. Remote Sens..

[31] Chen S., Lu Z., Xu X., Liu J., Bi Z. (2022). Foot-mounted Dual-sensor Single-board Pedestrian Inertial Navigation System Based on Position and Velocity Constraints. Sens. Mater..

[32] Lee M.S., Park C.G., Ju H.J., Song J.W. Use of multiple wearable inertial sensors in human localization. Proceedings of the ION 2015 Pacific PNT Meeting.

[33] Lambrecht S., del-Ama A.J. (2014). Human movement analysis with inertial sensors. Emerging Therapies in Neurorehabilitation.

[34] Blanco C.M., Bouillard P., Bodarwe E., Ney L. Structural dynamic design of a footbridge under pedestrian loading. Proceedings of the 9th SAMTECH Users Conference.

[35] Ben Y., Yin G., Gao W., Sun F. Improved filter estimation method applied in zero velocity update for SINS. Proceedings of the 2009 International Conference on Mechatronics and Automation.

[36] Pham D.D., Suh Y.S. (2016). Pedestrian navigation using foot-mounted inertial sensor and LIDAR. Sensors.

[37] Mbalawata I.S., Särkkä S., Haario H. (2013). Parameter estimation in stochastic differential equations with Markov chain Monte Carlo and non-linear Kalman filtering. Comput. Stat..

[38] Colomar D.S., Nilsson J.-O., Händel P. Smoothing for ZUPT-aided INSs. Proceedings of the 2012 International Conference on Indoor Positioning and Indoor Navigation (IPIN).

[39] Simon D., Simon D.L. (2006). Kalman filtering with inequality constraints for turbofan engine health estimation. IEE Proc.-Control Theory Appl..

[40] Kim J., Suh T., Ryu J. Inequality constrained kalman filter for bearing-only target motion analysis. Proceedings of the 2015 15th international conference on control, automation and systems (ICCAS).

[41] Skog I., Handel P., Nilsson J.-O., Rantakokko J. (2010). Zero-velocity detection—An algorithm evaluation. IEEE Trans. Biomed. Eng..

